# Renal Metastatic Melanoma Presenting With Solitary Mass

**DOI:** 10.7759/cureus.51253

**Published:** 2023-12-28

**Authors:** Tuğçem Bıçak, Selver Ozekinci, Yekta Bıçak, Mansur Dağgülli

**Affiliations:** 1 Pathology, Sağlık Bilimleri Üniversitesi (SBÜ) Gazi Yaşargil Eğitim Ve Araştırma Hastanesi, Diyarbakır, TUR; 2 Pathology, Dicle University Faculty of Medicine, Diyarbakır, TUR; 3 Urology, Dicle University Faculty of Medicine, Diyarbakır, TUR

**Keywords:** solitary kidney mass, skin, kidney, metastasis, melanoma

## Abstract

The kidney is a rare site of metastatic implantation. Metastases to the kidney most commonly originate from carcinomas in the lungs, breasts, and colon. Melanoma metastasis to the kidney is rare. We present an unusual case of melanoma metastasis to the kidney arising in a 76-year-old male who was diagnosed with melanoma two years ago. We emphasize the importance of thorough patient anamnesis when diagnosing renal cell carcinoma or urothelial carcinoma is challenging. In cases where patients with a history of melanoma present with new masses or lesions, even in atypical areas, considering melanoma metastasis in the differential diagnosis is crucial.

## Introduction

Metastases to the kidney most commonly originate from carcinomas in the lungs, breasts, and colon. Renal metastases from melanoma are rare. Renal metastasis of melanoma usually occurs in end-stage disease [1]. Solid organ metastases in the kidney are usually multiple and bilateral. In the presence of a solitary renal mass, many lesions, primarily primary renal neoplasms, are included in the differential diagnosis. After the patient was diagnosed with melanoma, it was learned that he was diagnosed with melanoma from the skin of his arm two years ago through the information provided by the Türkiye state health system. The history of melanoma was initially obscured at the time of microscopic examination, and this history only came to light subsequently. Thus, a solitary mass in the renal hilus was diagnosed as melanoma metastasis. We wanted to share our experiences in this rare case.

## Case presentation

A 76-year-old male was admitted to the urology outpatient clinic with complaints of intermittent right flank pain, intermittent hematuria, dysuria, pollakiuria, and nocturia. Radiological examination revealed a tumor mass with spiculated contours, 11 cm in largest diameter, obliterating the ureteral lumen. The mass was located in the pelvis of the right kidney.

**Figure 1:**
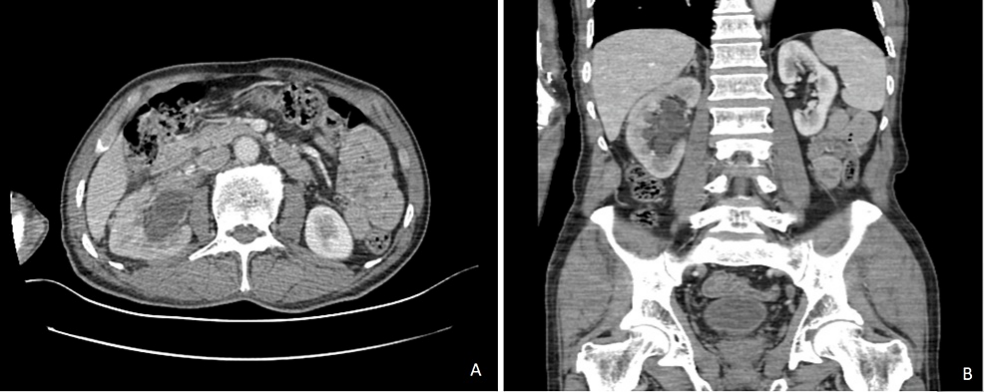
CT scan showing a spiculated contoured mass in the right kidney. (A) Axial plana, (B) coronal plane

Radiological findings were compatible with renal urothelial carcinoma (Figure 1). The results of routine blood testing (complete blood count, basic metabolic panel) were within the normal ranges.

**Figure 2:**
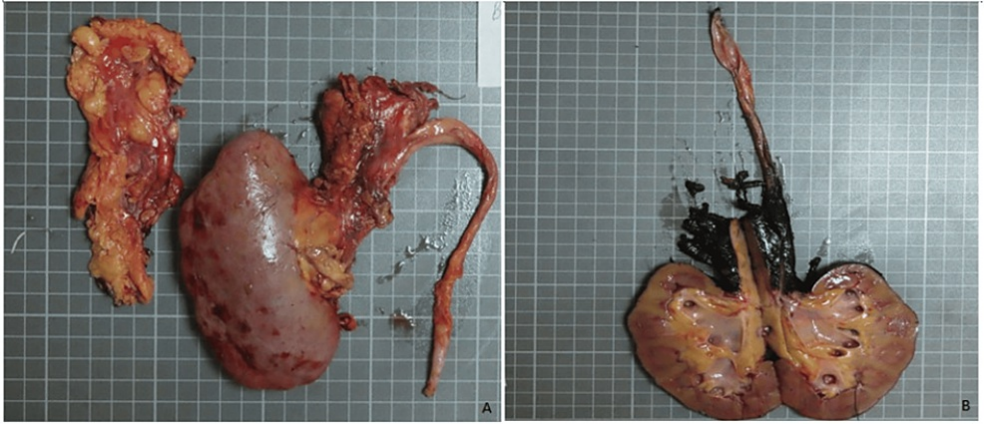
Macroscopic image of the tumor extending outside the kidney from the calyces, located in the renal pelvis. (A) Nephrectomy specimen and accompanying fat tissue. (B) An incision was made on the antihilar side of the specimen Note: Each of the small squares in the background measures 1 x 1 cm

The patient underwent a right radical nephrectomy. A mass measuring 11 x 6 x 1.8 cm was found in the renal pelvis, calyces, and ureter, protruding from the calyx and the peripelvic fat. On cut sections, the mass showed a tan-pale solid texture. No macroscopic lesion was seen in the uninvolved kidney (Figure 2).

**Figure 3:**
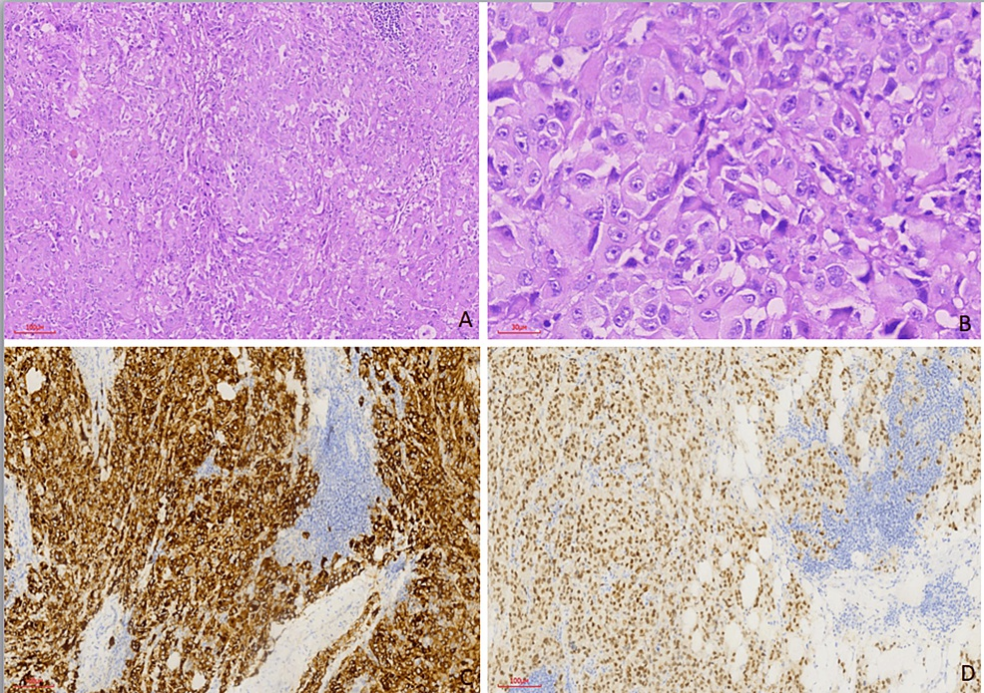
(A) H&E (100x). (B) H&E (400x), tumoral cells with large epithelioid cytoplasm and prominent nucleoli, positive with (C, D) MelanA and PRAME antibody (100x)

Formalin-fixed and paraffin-embedded tissue sections were examined under light microscopy. Microscopic examination revealed that the tumor consisted of large epitheloid cells, some of which contained brown pigments and prominent nucleoli. Frequent mitosis was noted (Figure 3).

Immunostaining showed that the tumor cell was positive for HMB-45 (Ventana, mouse antibody), SOX10 (Ventana, rabbit antibody, clone SP267), PRAME (Ventana, rabbit antibody, clone EPR20330), MelanA (Ventana, mouse antibody, clone A103), and S100 (Ventana, mouse antibody, clone 4C4.9), and negative for pancytokeratin AE1/AE3 (Ventana, mouse antibody, clone AE1/AE3/PCK26), cytokeratin 7 (SP52), pax8 (Ventana, mouse antibody, clone MRQ-50), TFE3 (Cell Marque rabbit antibody, clone MRQ-37), GATA3 (Ventana, mouse antibody, clone L50-823), and P63 (Ventana, mouse antibody, clone 4A4). This immunostain profile was diagnostic for melanoma. It was learned that the patient was diagnosed with melanoma on the skin of the arm two years prior in the Türkiye state health system. Therefore, the diagnosis was revised to metastatic melanoma. After the diagnosis of metastatic melanoma was made, PET-CT was performed. PET images from two years ago were taken from the Türkiye state healthcare system. Two years ago, there were metastases in the cervical lymph nodes on PET-CT. There was no involvement in the pelvis or abdomen. Current PET-CT showed metastasis in cervical lymph nodes, including submandibular, mediastinal lymph nodes, bilateral lungs, and thoracic 4 vertebra. The patient received chemotherapy and high-dose interleukin-2 treatment but did not respond and died eight months later.

## Discussion

In the literature, the rate of microscopic metastasis of melanoma at autopsy has been reported to reach 50% [2]. Malignancies from the stomach, lung, and breast are the most common metastasizers to the kidney. Melanoma metastasis is very rare. In approximately 4% of patients with metastatic melanoma, the primary lesion cannot be found [2].

The differential diagnosis includes tumors that can produce melanin and/or express melanocytic markers, such as melanin-producing perivascular epithelioid cell tumor (PEComa), melanin-pigmented renal cell carcinoma, and translocated renal cell carcinoma [3]. In melanin-pigmented renal cell carcinoma, the cytoplasm of tumor cells is clear, and melanocytic markers are negative. In contrast to the clear cytoplasm of renal cell carcinoma, our case consisted of cells with eosinophilic cytoplasm and prominent nucleoli. PEComa shows numerous growth patterns, with sheets and nests being the most common, and contains non-cohesive epithelioid cells with clear to eosinophilic granular cytoplasm. PEComa may have prominent nucleoli, intranuclear pseudoinclusions, multinucleated cells, and melanin pigment (rare). It may be confused with melanoma. Characterized by thin and delicate vessels. Although morphologically similar to melanoma, it is differentiated by immunohistochemistry. PEComa, like melanoma, is HMB45 and MelanA positive. A positive for SMA and usually a negative for S-100 is helpful in the differential diagnosis. Melanoma is negative for smooth muscle markers such as SMA. MiT family translocation renal cell carcinoma also often has psammoma bodies and may contain melanin pigment, resembling melanoma. MelanA and HMB45 positivity may cause difficulty in differential diagnosis, but typical histopathological features (papillary and solid alveolar growth pattern, composed of clear to eosinophilic discohesive pseudostratified cells with voluminous cytoplasm and high-grade nuclei) observed in MiT translocation carcinomas are helpful in differential diagnosis [4]. When melanin pigment is not observed in the tumor, sarcomas such as high-grade, dedifferentiated clear cell sarcoma (CCS), angiosarcoma, and leiomyosarcoma should also be considered in the differential diagnosis. The fact that melanocytic markers such as MelanA and HMB5 are positive in CCS of soft tissue and members of the perivascular epithelioid cell tumor (PEComa) family such as angiomyolipoma creates difficulty in the differential diagnosis. The PRAME antibody is partially helpful. It is important to keep in mind that this immune profile can also be seen in CCS, with the difference that the PRAME is negative in 70% of cases. Also, the morphology is not of clear cell appearance. Still, CCS can present in a diffuse pattern with solid sheets of epithelioid to spindle cells. While PRAME negativity has been reported in CCSs in the literature, its positivity has been shown in melanoma metastases and the PEComa family [5,6].

Abeshouse reviewed 142 cases of melanoma in the genitourinary tract, including 86 secondary and 56 primary melanomas [1]. It has been observed that in 80% of melanoma metastases to the kidney, the primary lesion is from the skin [1]. Most kidney metastases were asymptomatic, disclosed at autopsy, and less than 1 cm [1]. Therefore, the presented case is unusual in that the tumor was symptomatic and large (11 cm) [1]. The initial symptoms in these reports were intermittent right flank pain, hematuria, dysuria, pollakiuria, and nocturia. Melanoma with any visceral metastasis usually has a poor prognosis; one-year survival is approximately 50% and two-year survival is approximately 25% [2].

The primary lesion may be clinically undetected or may have spontaneously regressed. Generally, renal metastases from disseminated melanoma are bilateral, multiple, less than 1 cm, and subcortical [2]. Since melanoma metastasis to the kidney is a rare entity, it is usually not at the top of the list of differential diagnoses of renal tumors. In this case, the patient's CT, at first glance, suggests renal cell carcinoma or urothelial carcinoma of the renal pelvis. The definitive diagnosis is usually made by careful examination of the histomorphology of the tumor and supported by immunohistochemical studies [3]. Melanomas of the kidney are metastatic; thus, careful examination of the histologic features together with evaluation of clinical data and the patient's imaging findings is required.

The optimal treatment for melanoma is surgical resection. Many adjuvant therapies are available, including chemotherapy such as fotemustine and dacarbazine and immunotherapy such as interferon-alpha and IL-2 [2]. Immunotherapy and/or chemotherapy are available treatment options for melanoma with multiple renal metastases or unresectable lesions, as in the present case [2].

## Conclusions

We emphasize the importance of thorough patient anamnesis when diagnosing renal cell carcinoma or urothelial carcinoma is challenging. In cases where patients with a history of melanoma present with new masses or lesions, even in atypical areas, considering melanoma metastasis in the differential diagnosis is crucial.
